# Radiation-Induced Meningiomas: Systematic Review with Pooled Case Analysis and Case Series of Long Latency, Aggressive Behavior, and Clinical Outcomes

**DOI:** 10.3390/jcm15062356

**Published:** 2026-03-19

**Authors:** Anastasija Krzemińska, Jakub Więcław, Marta Koźba-Gosztyła, Bogdan Czapiga

**Affiliations:** 1 Department of Neurosurgery, Wroclaw 4th Military Clinical Hospital, Weigla 5, 50-981 Wroclaw, Poland; 2 Faculty of Medicine, Wroclaw University of Science and Technology, Grunwaldzki Square 11, 51-377 Wroclaw, Poland

**Keywords:** radiation-induced, radiation-associated, secondary, meningioma

## Abstract

**Objective**: Radiation-induced meningiomas (RIMs) are a rare but clinically relevant late complication of cranial irradiation, characterized by long latency and potentially aggressive behavior. This study aimed to systematically analyze the relationships between radiation dose, age at irradiation, latency period, histological grade, tumor multiplicity, and recurrence in RIMs. **Methods**: A systematic review and pooled case analysis of published cases of RIMs was performed, supplemented by a case series of four institutional patients. Data were extracted on primary tumor type, radiation dose, age at irradiation, latency period, World Health Organization (WHO) grade, tumor multiplicity, and recurrence. Radiation dose was categorized as low (<20 gray (Gy)), intermediate (20–40 Gy), or high (>40 Gy). Statistical analyses included χ^2^ tests, Mann–Whitney U tests, Kruskal–Wallis tests, and Spearman correlation analyses. **Results**: A total of 1809 patients were included. A higher radiation dose was significantly associated with shorter latency (*p* < 0.001), a higher WHO grade (*p* < 0.001), and increased tumor multiplicity (*p* < 0.001). High-grade RIMs occurred predominantly after high-dose irradiation. Tumor recurrence was significantly more frequent in high-grade than low-grade meningiomas (51.5% vs. 18.3%, *p* < 0.001), but it was not associated with radiation dose. Older age at irradiation correlated with longer latency (Spearman’s ρ = 0.405, *p* < 0.001). No association was observed between primary tumor category and WHO grade. **Conclusions**: RIMs demonstrate dose- and age-dependent biological behavior, with higher radiation doses and younger age at irradiation predisposing to earlier onset and increased aggressiveness. These findings suggest that long-term, dose-adapted radiological surveillance may warrant consideration in irradiated patients.

## 1. Introduction

Meningiomas are the most common primary intracranial tumors in adults, accounting for approximately 30–40% of all brain neoplasms. In most cases, meningiomas are sporadic and benign (World Health Organization (WHO) grade I), although atypical and anaplastic forms (WHO grades II and III) account for a smaller but clinically important proportion of cases and are associated with higher recurrence rates and less favorable outcomes. Meningiomas arise from arachnoid cap cells and are associated with genetic alterations such as NF2, TRAF7, KLF4, or AKT1 mutations. However, in a small subset of patients, meningiomas may develop as a late consequence of previous exposure to ionizing radiation—whether diagnostic, therapeutic, or environmental [1,2].

Radiation-induced meningiomas (RIMs) represent a rare but well-recognized long-term complication of cranial irradiation. They were first described in the mid-20th century among patients who underwent scalp irradiation for tinea capitis. Today, RIMs are most frequently observed in individuals who received cranial radiotherapy during childhood or adolescence for primary central nervous system tumors, leukemia, or lymphoma.

Compared with sporadic meningiomas, RIMs tend to occur at a younger age, after a latent period ranging from 5 to over 30 years, and often present as multiple lesions. They also display a higher incidence of atypical or anaplastic histology (WHO grades II–III), increased recurrence rates, and more aggressive biological behavior. Surgical management can be challenging due to radiation-induced tissue fibrosis and adhesions within previously irradiated fields [3].

The pathogenesis of RIMs remains incompletely understood. Proposed mechanisms include radiation-induced DNA double-strand breaks, chromosomal instability, and the loss of tumor suppressor genes such as NF2 [4]. Advances in molecular profiling have revealed distinct mutational signatures in RIMs compared with sporadic meningiomas, suggesting a unique oncogenic pathway triggered by ionizing radiation.

Given the increasing number of long-term survivors of childhood and adult cancers, the clinical relevance of RIMs continues to grow. Although RIMs have been recognized for decades, the literature remains dominated by isolated case reports and small case series, with substantial heterogeneity in the reporting of radiation dose, latency, histological grade, multiplicity, and recurrence. As a result, clinically relevant questions regarding dose-dependent behavior, age-related risk, and long-term surveillance remain insufficiently addressed in large pooled analyses.

Although several systematic reviews have previously described RIMs, most were limited by smaller datasets and a lack of integrated analyses of latency, radiation dose, age at irradiation, tumor multiplicity, and histological grade. The present study provides one of the largest pooled analyses of reported RIM cases to date and explores the relationships between radiation exposure parameters and key clinical characteristics of these tumors. The aim of the present study is to perform a systematic review and pooled case analysis of all available cases of RIMs reported in the literature and to present four additional cases from our own institution. By integrating data from previously published reports and our clinical experience, this study seeks to better characterize the epidemiological and pathological features of RIMs and to compare them with sporadic meningiomas in terms of latency, histological grade, recurrence, and clinical outcomes.

## 2. Materials and Methods

### 2.1. Design of the Study

This study consisted of two complementary components: (1) a systematic literature review and pooled analysis of reported cases of RIMs and (2) a case series including four patients diagnosed and treated for RIMs at our institution.

Ethical approval was not required for the pooled case analysis as it was based exclusively on previously published data. Informed consent was obtained from all living patients included in the case series.

### 2.2. Case Reports

Four patients with histologically confirmed meningiomas arising within previously irradiated fields were identified at our neurosurgical department between 2017 and 2025. Demographic, clinical, radiological, and histopathological data were collected from institutional medical records. The following variables were analyzed for each case: sex, age at primary irradiation, indication for radiation therapy, total radiation dose, latency period between radiotherapy and tumor diagnosis, tumor location, radiological features, histological grade (according to the 2021 WHO Classification of Central Nervous System Tumors), treatment modality, recurrence, and clinical outcome.

The institutional cases were included as an exploratory validation cohort to illustrate the clinical spectrum of RIMs observed in contemporary practice.

### 2.3. Literature Review: Search Strategy and Data Extraction

A systematic search of the PubMed, Scopus, and Embase databases was performed up to 1 November 2025.

The search strategy included combinations of the following keywords and Medical Subject Headings (MeSH) terms:

(“radiation-induced meningioma” OR “radiation-associated meningioma” OR “secondary meningioma” OR “meningioma after radiotherapy”) AND (radiotherapy OR irradiation OR radiation exposure).

The search was limited to studies involving human subjects and articles published in English. No restrictions on publication year were applied.

The reference lists of all retrieved papers were also manually screened to identify additional eligible publications.

Studies were included if they reported individual or aggregated clinical data on meningiomas fulfilling the modified Cahan criteria for radiation-induced tumors: (1) the tumor developed within a previously irradiated field; (2) there was a sufficient latency period between irradiation and tumor appearance; (3) the histology of the secondary tumor differed from that of the primary lesion, and (4) the patient had no predisposing conditions such as neurofibromatosis type 2, Li–Fraumeni syndrome, or Gorlin syndrome. We also excluded patients with retinoblastoma as a primary disease because of the reported germline pathogenic variants of the RB1 gene in about 40% of patients with retinoblastoma [5]. Although DNA damage resulting from radiotherapy occurs almost immediately after exposure, the minimum duration of a sufficient latency period remains undefined, and reported intervals vary widely across published studies. We included RIMs with > 6-month latency periods.

Two reviewers independently screened titles and abstracts and extracted data from eligible full-text articles using a standardized data collection form. The extracted variables included patient age, sex, primary disease, radiation dose and field, latency period, tumor multiplicity, anatomical location, WHO grade, treatment modality, recurrence, and survival data when available.

This study followed Preferred Reporting Items for Systematic Reviews and Meta-Analyses (PRISMA) guidelines for systematic reviews; however, due to the descriptive and case-based nature of the available literature, the quantitative synthesis was conducted as a pooled case analysis rather than a conventional study-level meta-analysis.

### 2.4. Selection Criteria

Eligible studies included case reports, case series, and retrospective cohort studies, providing sufficient clinical and histological information to confirm the diagnosis of RIM and to extract at least one of the following data points: primary tumor type, latency period, meningioma, irradiation dose, histological grade, recurrence, or survival. Reviews, editorials, conference abstracts, and experimental studies without individual clinical data were excluded. When multiple publications described overlapping cohorts, only the most comprehensive or recent study was included.

### 2.5. Outcome Measures

The primary outcomes included the latency period (interval between radiation exposure and meningioma diagnosis), histological grade, primary tumor type, age at meningioma diagnosis, radiation dose, and lesion multiplicity.

Secondary outcomes consisted of analyses of the relationships between primary tumor type, radiation dose, and age at irradiation and the latency period, meningioma WHO grade, and tumor multiplicity. Secondary analyses were exploratory in nature and were performed on variable-specific subsets of cases with complete data.

Primary tumors treated with radiotherapy were analyzed using predefined diagnostic categories to reduce heterogeneity and enable subgroup analyses. Individual diagnoses were grouped based on the anatomical location and clinical indication for radiotherapy (e.g., pituitary neuroendocrine tumors and craniopharyngiomas were classified as sellar/suprasellar tumors). Rare, heterogeneous, or insufficiently specified diagnoses that could not be reliably assigned to a specific category were grouped as “Other.”

### 2.6. Subgroup Datasets

Due to heterogeneity and incomplete reporting in the available literature, analyses were performed on variable-specific subsets of cases. For each sub-analysis, only patients with complete data for the variables of interest were included. Consequently, the number of cases differed between analyses.

### 2.7. Statistical Analysis

All statistical analyses were performed using Statistica (TIBCO Software, version 13.1.0, StatSoft GmbH, Hamburg, Germany).

Because the available literature consisted predominantly of case reports, case series, and retrospective descriptive cohorts with heterogeneous reporting, the present study was designed as a pooled analysis of published cases rather than a conventional study-level meta-analysis of comparative effect estimates.

Continuous variables were assessed for distributional characteristics and were summarized as mean ± standard deviation (SD) and/or median with interquartile range (IQR) and range, depending on normality and data dispersion. Categorical variables were presented as counts and percentages.

For comparisons of continuous variables between more than two independent groups (e.g., latency period across primary tumor categories or radiation dose categories), the Kruskal–Wallis test was used. For two-group comparisons (e.g., age at irradiation in low-grade vs. high-grade RIMs), the Mann–Whitney U test with continuity correction was applied. Associations between categorical variables (e.g., the WHO grade category vs. the radiation dose category; multiplicity vs. the radiation dose category; and recurrence vs. the WHO grade category) were evaluated using Pearson’s chi-square test. When applicable, alternative chi-square estimates (including Yates correction) and Fisher’s exact test were reviewed for consistency in 2 × 2 tables.

Correlation between continuous variables (e.g., age at irradiation and latency period) was assessed using Spearman’s rank correlation coefficient (ρ). All statistical tests were two-tailed, and a *p*-value < 0.05 was considered statistically significant.

To account for studies reporting aggregated data (i.e., multiple cases summarized in a single row), analyses were weighted by the reported number of cases in order to approximate patient-level distributions and avoid underrepresentation of larger cohorts.

Because the study did not pool study-level comparative effect sizes, conventional fixed-effect or random-effects meta-analytic models, heterogeneity statistics (I^2^ or τ^2^), forest plots, and publication bias analyses were not methodologically appropriate and were therefore not performed.

Graphical visualizations were generated using Microsoft Excel (Microsoft Corporation, Redmond, WA, USA).

Kaplan–Meier analysis for latency period was not performed, as the available literature consisted of case-based observations without censoring and without a defined time-to-event framework at the cohort level.

### 2.8. Quality Assessment

The methodological quality of the included studies was assessed using the Joanna Briggs Institute (JBI) Critical Appraisal Checklists for Case Reports and Case Series (Supplementary Table S1). Each study was independently evaluated by two reviewers, and discrepancies were resolved by consensus.

## 3. Results

### 3.1. Case Series

#### 3.1.1. Case 1 (2007, 2013, and 2022)

A male patient with a history of right adrenal and upper mediastinal neuroblastoma was treated at the age of 2 years with surgical resection followed by combined chemotherapy and radiotherapy. Due to intracranial metastatic disease, high-dose cranial irradiation was administered.

Twenty years later, the patient developed a meningioma within the previously irradiated field and underwent surgical resection. The tumor recurred five years later and was resected again, followed by a second recurrence nine years thereafter, which also required surgical treatment.

Histopathological examination revealed an atypical meningioma (CNS WHO grade II). The postoperative course was uneventful. The patient remains free from tumor recurrence to date.

#### 3.1.2. Case 2 (2017 and 2019)

A male patient with acute lymphoblastic leukemia diagnosed at the age of 2.5 years was treated with multimodal therapy, including cranial radiotherapy. Definitive treatment was completed with allogeneic bone marrow transplantation at the age of 12 years.

Twenty-four years later, the patient developed recurrent frontal headaches. Neuroimaging revealed a large frontal extra-axial tumor measuring approximately 110 × 100 mm. Surgical resection was performed. Due to tumor recurrence, a second surgical procedure was carried out two years later.

**Figure 1:**
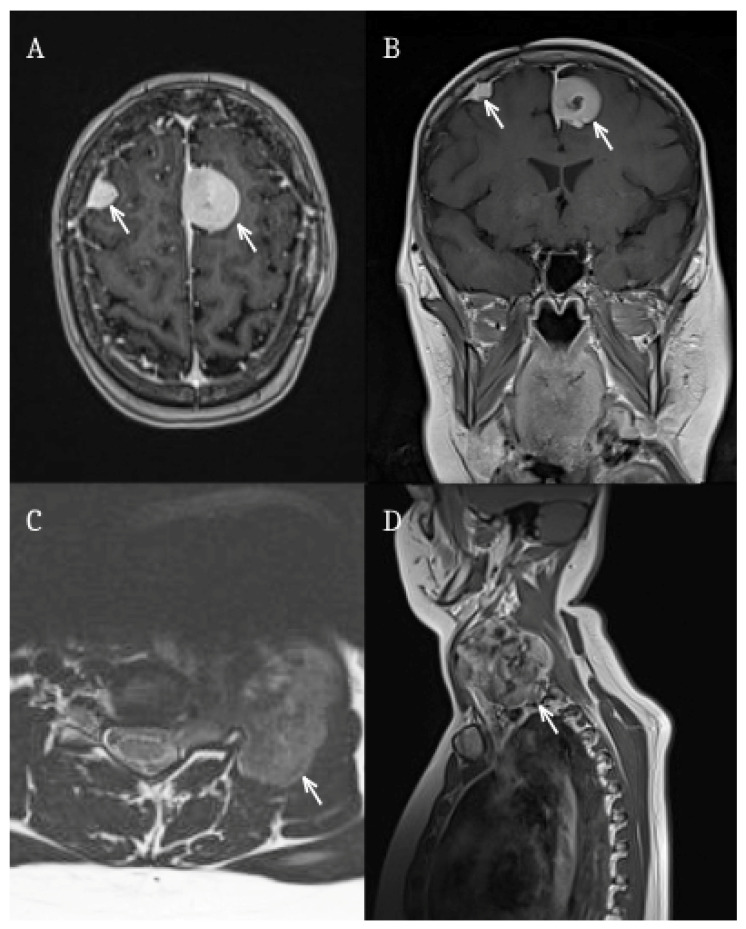
Magnetic resonance imaging findings in Patient 2 with RIMs. White arrows indicate the tumors. (**A**) Axial T1-weighted post-contrast image demonstrating multifocal intracranial meningiomas. (**B**) Coronal T1-weighted post-contrast image confirming multiple contrast-enhancing lesions. (**C**) Axial MRI of the cervical region showing a large extracranial mass suspected to represent a meningioma. (**D**) Sagittal MRI of the cervical spine demonstrating the extent of the cervical lesion.

In both surgeries, histopathological examination confirmed a fibroblastic meningioma (CNS WHO grade I). During follow-up, further recurrence was detected, with two intracranial meningiomas currently managed conservatively under radiological surveillance (Figure 1A,B). Additionally, a large cervical mass has been identified and is scheduled for priority surgical treatment; based on imaging characteristics, the lesion is suspected to represent an extracranial meningioma (Figure 1C,D).

#### 3.1.3. Case 3 (2023)

A male patient with a history of acute lymphoblastic leukemia was treated with cranial radiotherapy at the age of 7 years. Thirty-one years later, he presented with progressive headaches, visual disturbances, and mild paresis of the left abducens nerve.

**Figure 2:**
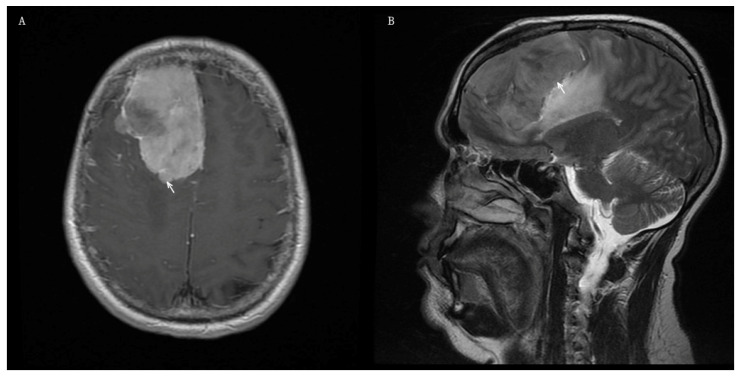
Preoperative MRI of Patient 3 demonstrating a RIM located in the right frontal lobe. White arrows indicate the tumors. (**A**) Axial T1-weighted image after gadolinium administration showing intense contrast enhancement of the lesion. (**B**) Sagittal T2-weighted image demonstrating peritumoral brain edema.

Brain MRI revealed a large, irregular, contrast-enhancing parasagittal mass in the right frontal lobe measuring 65 × 48 × 62 mm (Figure 2A,B). A right frontal craniotomy was performed, and gross total resection of the tumor was achieved.

Histopathological examination confirmed a meningothelial meningioma (CNS WHO grade I). The postoperative course was uneventful, and the patient remains free from tumor recurrence to date.

#### 3.1.4. Case 4 (2025)

A male patient with a history of right cingulate gyrus ependymoma was treated with surgical resection followed by adjuvant radiotherapy at the age of 11 years.

Twenty-nine years later, the patient developed progressive weakness of the left upper and lower extremities, intermittent divergent strabismus of the right eye, occasional horizontal nystagmus on right gaze, and mild left facial weakness.

**Figure 3:**
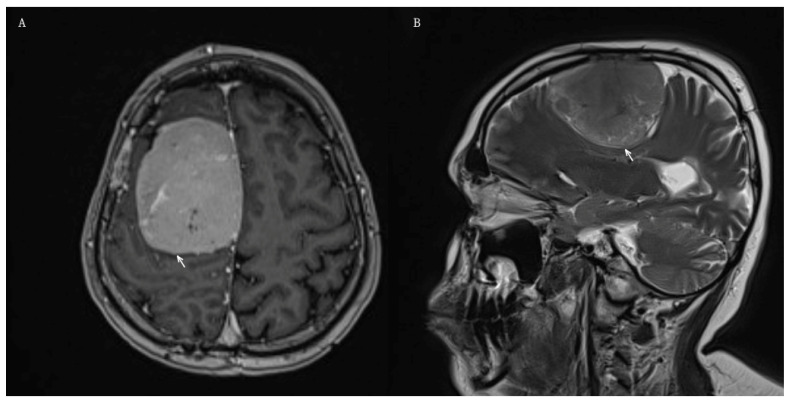
Preoperative MRI of Patient 4 demonstrating a RIM located in the right cerebral convexity. White arrows indicate the tumors. (**A**) Axial T1-weighted post-contrast image showing homogeneous enhancement of the tumor. (**B**) Sagittal T2-weighted image.

Brain MRI demonstrated a large parasagittal extra-axial tumor located on the right cerebral convexity, measuring 67 × 52 × 52 mm (Figure 3A,B). The patient underwent a right parietofrontal craniotomy with near-total tumor resection (Simpson grade III).

Histopathological examination confirmed an atypical meningioma (CNS WHO grade II). Postoperatively, neurological improvement was observed, along with regression of left-sided hemiparesis. A multidisciplinary oncology board recommended postoperative radiotherapy. The patient remains free from tumor recurrence to date.

### 3.2. Search Results

**Figure 4:**
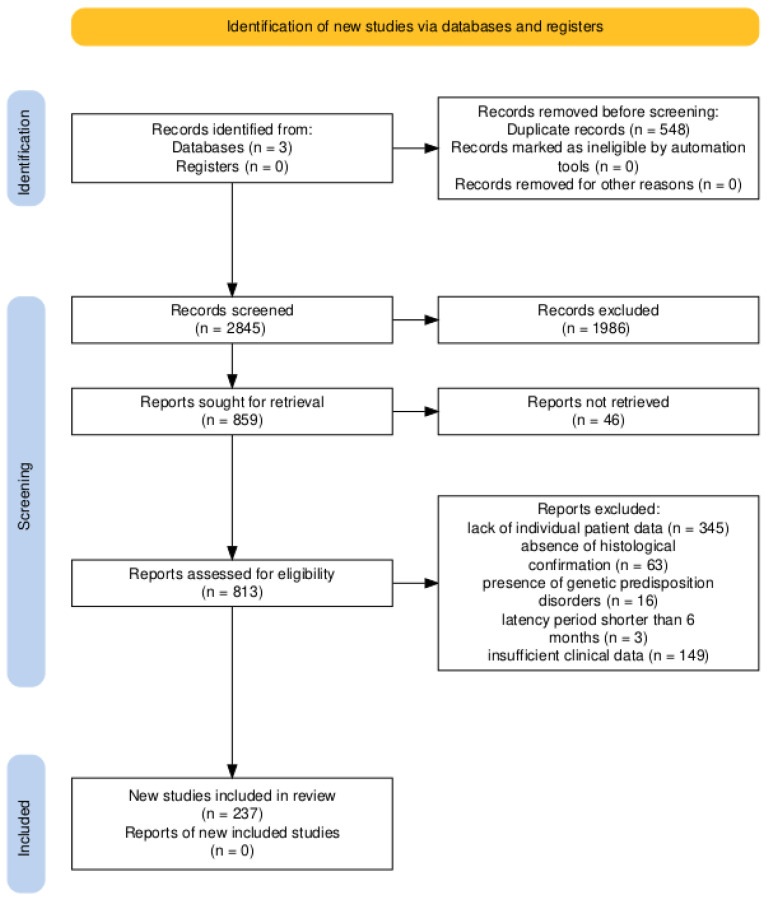
Flow diagram illustrating the identification, screening, eligibility assessment, and inclusion of studies reporting RIMs in the systematic review and meta-analysis. From Haddaway et al. [6].

The study selection process is summarized in the PRISMA flow diagram (Figure 4).

A total of three databases were searched, yielding 3393 records. After removal of 548 duplicate records, 2845 records were screened based on titles and abstracts, of which 1986 were excluded.

Full-text articles were sought for 859 reports, but 46 reports could not be retrieved. The remaining 813 full-text articles were assessed for eligibility. Of these, 578 reports were excluded for the following reasons: lack of individual patient data (*n* = 345), absence of histological confirmation (*n* = 63), presence of genetic predisposition disorders (*n* = 16), latency period shorter than 6 months (*n* = 3), or insufficient clinical data (*n* = 149).

Ultimately, 237 studies met the inclusion criteria and were included in the systematic review and pooled case analysis. The included studies were published between 1953 and 2025. A list of all studies included in the meta-analysis is presented in Supplementary Table S2.

The four cases from our institution were analyzed separately as an exploratory institutional validation cohort and were not included in the pooled analysis of previously published cases. The clinical characteristics of the four institutional cases are summarized in Table 1.

### 3.3. Baseline Characteristics

A total of 1809 patients with RIMs were included in the pooled case analysis. Due to incomplete and heterogeneous reporting across the included studies, baseline characteristics were limited. Sex distribution was the only variable consistently reported for the majority of cases: 522 patients were male, while 642 were female, and sex was not specified in 645 cases. Other clinical variables were analyzed in variable-specific subgroups based on data availability and are presented in subsequent analyses.

### 3.4. Sub-Analyses

#### 3.4.1. Data Availability

Due to heterogeneous and incomplete reporting across the included studies, the availability of clinical variables varied substantially. Data availability for variables included in the subgroup analyses is summarized in Table 2. Subgroup analyses evaluating the associations between primary tumor categories and latency period, histological grade, radiation dose, and tumor multiplicity were performed on variable-specific subsets of cases with available data and are presented separately.

##### Primary Tumor Categories

Data on the primary tumor treated with radiotherapy were available for 1746 cases. The most frequent primary indication for radiotherapy was skin disease of the head, accounting for 530 cases (48.1%). This category included both benign and malignant dermatological conditions historically treated with irradiation, such as tinea capitis, cutaneous hemangiomas, and scalp malignancies. Acute leukemia (acute lymphoblastic leukemia and acute myeloid leukemia) represented the second most common category, comprising 378 cases (34.3%). The high proportion of RIMs observed after acute leukemia reflects historical treatment strategies, in which prophylactic cranial irradiation was routinely administered in pediatric patients to prevent central nervous system relapse. Cranial irradiation doses of 18–24 gray (Gy) directly exposed the meninges to ionizing radiation, predisposing long-term survivors to the development of secondary meningiomas decades later.

Among central nervous system tumors, medulloblastoma was the most frequent primary diagnosis (137 cases, 12.4%), followed by low-grade glioma (98 cases, 8.9%) and sellar or suprasellar tumors, including pituitary neuroendocrine tumors and craniopharyngiomas (62 cases, 5.6%). Less common categories included non-central nervous system lymphomas (39 cases, 3.5%), ependymomas and subependymomas (33 cases, 3.0%), and germ cell tumors (19 cases, 1.7%).

Rare primary tumor categories, each accounting for less than 1% of cases, included head and neck tumors, high-grade gliomas, other central nervous system embryonal tumors, primitive neuroectodermal tumors, pineal tumors, ventricular tumors, and posterior fossa tumors other than medulloblastomas.

Exposure related to nuclear incidents, including survivors of the atomic bomb explosion in Nagasaki and individuals exposed to the Chernobyl nuclear accident, accounted for 204 cases (18.5%). Other heterogeneous or insufficiently specified conditions that could not be reliably assigned to a specific category were grouped as “Other” and included 180 cases (16.3%).

The complete classification scheme and corresponding entities are presented in Table 3.

1. Primary tumor category and latency period

A weighted Kruskal–Wallis test demonstrated a statistically significant difference in latency period among primary tumor categories (H = 297.8, df = 8, and *p* < 0.001). The analysis included 815 patients with available latency data. Longer latency periods were observed in patients irradiated for skin diseases of the head and in victims of nuclear incidents, whereas shorter latency periods were associated with medulloblastoma and non-CNS lymphoma. Detailed latency distributions by primary tumor category are presented in Table 4.

2. Primary tumor category and WHO grade of RIMs

The association between primary tumor category and meningioma WHO grade (low-grade vs. high-grade) was assessed using the χ^2^ test in a case-weighted analysis. A statistically significant association was observed (χ^2^ = 29.26, df = 8, and *p* = 0.0029; Cramér’s V = 0.29). The analysis included 347 cases with available RIM WHO grade data. The proportion of high-grade meningiomas varied across primary tumor categories, with the highest in the ependymoma/subependymoma group (41.7%) and the lowest among victims of nuclear incidents (4.5%). Detailed results are presented in Table 4.

3. Primary tumor category and RIM tumor multiplicity

A significant association was observed between primary tumor category and tumor multiplicity (χ^2^(8) = 238.4; *p* < 0.001), with multifocal meningiomas occurring more frequently after irradiation for ependymoma/subependymoma. Data on tumor multiplicity and primary tumor category were available for 688 cases. Detailed results are presented in Table 4.

##### Dose of Irradiation

Radiation dose was categorized as follows: low dose (<20 Gy), intermediate dose (20–40 Gy), and high dose (>40 Gy).

Data on radiation dose were available for 1478 cases. Of these, 590 cases (39.9%) received low-dose radiotherapy, 226 cases (15.3%) received intermediate-dose radiotherapy, and 662 cases (44.8%) received high-dose radiotherapy.

1. Radiation dose and latency period

Data on latency period and the radiation dose category were available for 1130 cases.

**Figure 5:**
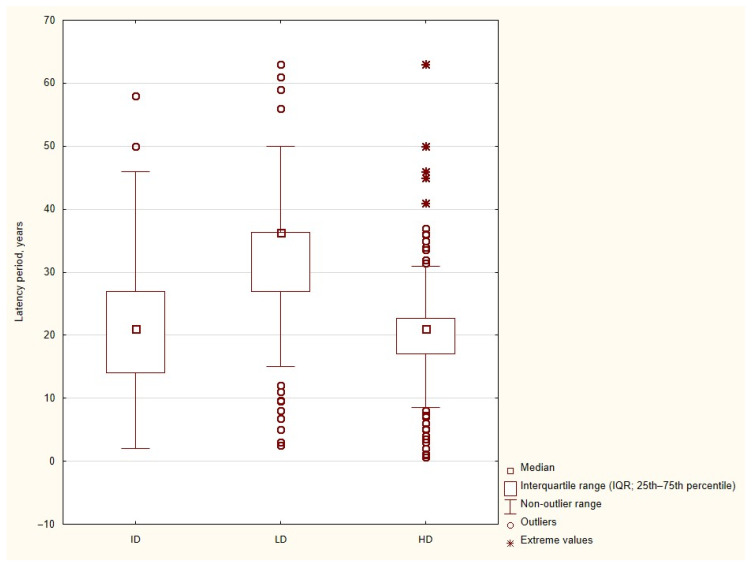
Latency period according to the radiation dose category in RIMs. Legend: Box-and-whisker plot showing the distribution of latency period (years) of RIMs according to the radiation dose category: low dose (<20 Gy), intermediate dose (20–40 Gy), and high dose (>40 Gy). Boxes represent the interquartile range (IQR; 25th–75th percentile), while horizontal lines indicate the median, and whiskers denote the non-outlier range. Circles indicate outliers, and asterisks represent extreme values. To account for aggregated data reported in the literature, individual records were replicated according to the reported number of cases, allowing visualization of patient-level weighted distributions.

There was a statistically significant association between the radiation dose category and latency period (Kruskal–Wallis test: H = 299.35, df = 2, and *p* < 0.001). Patients treated with low-dose radiotherapy had the longest latency period, with a median latency of 36.3 years (IQR: 27.0–36.3; range: 2.5–63 years; *n* = 458). In contrast, patients exposed to high-dose radiotherapy developed RIMs after a significantly shorter latency, with a median of 21.0 years (IQR: 17.0–22.7; range: 0.6–63 years; *n* = 507) (see Figure 5). The intermediate-dose group showed a median latency of 21.1 years (IQR: 14.0–27.0; range: 2.0–58 years; *n* = 165). Overall, higher radiation doses were associated with shorter latency periods, suggesting a dose-dependent acceleration of RIM development.

2. Radiation dose and RIM multiplicity

Data on meningioma multiplicity overall were available for 1047 cases. Of these, 866 cases (82.7%) presented with a single lesion, whereas 181 cases (17.3%) had multiple lesions.

RIM multiplicity was significantly associated with the radiation dose category (χ^2^ = 16.70, df = 2, and *p* < 0.001).

Multifocal meningiomas were more frequently observed in patients exposed to high-dose radiotherapy compared with those treated with intermediate- and low-dose irradiation. Data on irradiation dose and tumor multiplicity were available for 808 cases. Detailed results are presented in Table 5.

##### RIM Recurrence

Tumor recurrence occurred in 109 of 440 cases (24.8%).

Tumor recurrence occurred significantly more often in high-grade meningiomas than in low-grade tumors (51.5% vs. 18.3%; χ^2^ = 26.61, df = 1, and *p* < 0.001). Data on tumor recurrence and RIM WHO grade were available for 241 cases. Detailed results are presented in Table 6.

No significant association was observed between the radiation dose category and tumor recurrence (χ^2^ = 1.55, df = 2, *p* = 0.46).

##### WHO Grade of RIM

Data on RIM WHO grade were available for 1017 cases. Of these, 702 cases (69%) were classified as WHO grade 1, 255 cases (25.1%) as WHO grade 2, and 60 cases (5.9%) as WHO grade 3.

**Figure 6:**
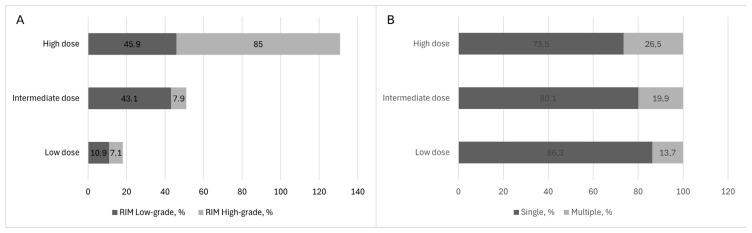
Association between radiation dose with WHO grade and tumor multiplicity in RIMs. (**A**) Distribution of WHO grade according to the radiation dose category. High-grade RIMs were more frequently observed in patients exposed to high-dose radiotherapy (>40 Gy). (**B**) Distribution of tumor multiplicity according to the radiation dose category. Multifocal meningiomas occurred more frequently after higher radiation doses. Radiation dose was categorized as low (<20 Gy), intermediate (20–40 Gy), and high (>40 Gy).

A higher radiation dose used for treatment of the primary tumor was associated with a higher WHO grade of RIM (χ^2^ = 119.1, df = 2, *p* < 0.001). High-grade RIMs developed predominantly in patients exposed to high-dose radiotherapy (85.0%), whereas low-grade tumors were more evenly distributed across dose categories. Data on irradiation dose and WHO grade were available for 806 cases. Detailed results are presented in Table 5 and Figure 6.

##### Latency Period

Data on latency period were available for 1303 cases. The median latency period was 22.7 years (range: 0.6–63 years), with a mean latency of 24.2 ± 10.9 years.

**Figure 7:**
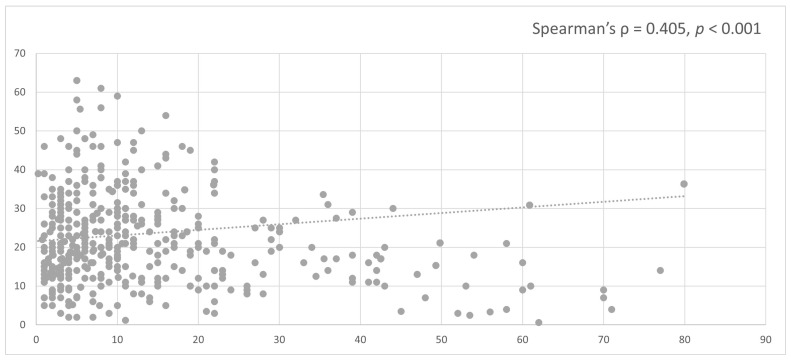
Correlation between age at irradiation and latency period in RIMs. Scatter plot illustrating the relationship between age at irradiation and the latency period (years) between radiation exposure and the diagnosis of RIM. Each dot represents an individual case. A significant positive correlation was observed, indicating that older age at the time of radiotherapy was associated with a longer latency period (Spearman’s ρ = 0.405; *p* < 0.001). The dashed line represents the linear trend.

A significant positive correlation was observed between age at irradiation and latency period (Spearman’s ρ = 0.405, *p* < 0.001), indicating that older age at the time of radiotherapy was associated with a longer latency to the development of RIM (see Figure 7).

##### Age at Irradiation

Data on age at irradiation were available for 980 cases. The median age at irradiation was 8.3 years, with a range from 0.3 to 79 years. The mean age was 34.7 ± 30.6 years, reflecting a markedly right-skewed distribution.

Age at irradiation differed significantly between RIM WHO grade groups. Patients who developed high-grade RIMs were irradiated at a significantly older age compared with those who developed low-grade tumors (Mann–Whitney U = 22,244; Z = 3.26; *p* = 0.001).

## 4. Discussion

RIMs represent a distinct clinical and biological entity compared with sporadic meningiomas. By integrating the largest pooled analytic dataset to date with an institutional case series, the present study provides a comprehensive overview of latency patterns, histological grade, multiplicity, recurrence, and their associations with radiation dose and age at irradiation. Several clinically relevant observations emerge from our analysis.

### 4.1. Radiation-Induced Meningiomas Versus Sporadic Meningiomas

In the general population, sporadic meningiomas are predominantly benign. According to large epidemiological and surgical series, approximately 75–80% of sporadic meningiomas are classified as WHO grade I, 15–20% as WHO grade II, and only 1–3% as WHO grade III [7,8]. Tumor multiplicity in sporadic meningiomas is relatively uncommon, reported in approximately 1–10% of cases, most often in association with neurofibromatosis type 2 [9]. Recurrence rates after gross total resection range from 5 to 10% for WHO grade I tumors and from 30 to 40% for WHO grade II tumors but exceed 50–70% for WHO grade III lesions [10,11,12].

In contrast, our pooled analysis confirms that RIMs exhibit a distinctly more aggressive phenotype. Although WHO grade I tumors still predominate, the proportion of high-grade lesions (WHO grades II–III) is markedly increased (25.1% WHO grade II and 5.9% WHO grade III) compared with sporadic meningiomas. Similarly, tumor multiplicity is substantially more frequent, affecting approximately 17% of cases in our cohort, far exceeding rates reported for sporadic diseases. Recurrence was observed in nearly one-quarter of cases with available follow-up data and occurred significantly more often in high-grade tumors, underscoring the aggressive biological behavior of RIMs.

These findings align with previous reports suggesting that radiation exposure not only initiates tumorigenesis but may also promote genomic instability, clonal heterogeneity, and malignant progression, resulting in tumors that behave more aggressively than their sporadic counterparts [13].

From a molecular perspective, RIMs are believed to differ from sporadic meningiomas, with prior studies suggesting distinct mutational patterns, chromosomal instability, and frequent alterations involving tumor suppressor pathways such as NF2. Although molecular data were unavailable for most cases included in the present analysis, the higher frequency of high-grade histology and recurrence in RIMs is consistent with the concept of radiation-driven genomic instability.

### 4.2. Primary Tumor Category and Histological Grade

Despite significant differences in latency period across primary tumor categories, no association was observed between the type of primary tumor treated with radiotherapy and the WHO grade of the resulting meningioma. This suggests that the histological aggressiveness of RIMs may be largely independent of the original disease for which radiotherapy was administered.

This observation supports the concept that radiation itself, rather than the biological characteristics of the primary tumor, is the dominant driver of malignant transformation in RIMs. Once a critical threshold of radiation-induced DNA damage is exceeded, downstream oncogenic pathways—such as chromosomal instability, loss of tumor suppressor genes (e.g., NF2), and accumulation of secondary mutations—may follow a relatively uniform trajectory regardless of the initial clinical indication for irradiation. This may explain why high-grade RIMs occur across diverse primary tumor categories, from benign scalp conditions to malignant central nervous system tumors.

### 4.3. Age at Irradiation and Latency Period

A key finding of the present study is the significant positive correlation between age at irradiation and latency period. Patients irradiated at a younger age developed RIMs after a shorter latency, whereas older age at exposure was associated with longer latency intervals.

Several biological mechanisms may underlie this relationship. First, the developing brain is characterized by higher cellular proliferation rates, increased stem and progenitor cell populations, and heightened vulnerability to DNA damage. Radiation exposure during childhood may therefore result in a larger pool of genetically altered precursor cells capable of malignant transformation. Second, DNA repair mechanisms in younger individuals, while efficient, may permit survival of damaged cells with misrepaired double-strand breaks, facilitating long-term clonal expansion. Third, the longer remaining lifespan after childhood irradiation allows sufficient time for additional genetic hits to accumulate, accelerating tumor development.

These factors collectively provide a plausible explanation for the shorter latency observed in patients irradiated at a young age and highlight childhood radiotherapy as a particularly strong risk factor for the development of RIMs.

### 4.4. Radiation Dose and Latency: Implications for Surveillance

Our data demonstrate a clear dose-dependent relationship between radiation exposure and latency period. High-dose radiotherapy (>40 Gy) was associated with significantly shorter latency, whereas low-dose exposure (<20 Gy) resulted in the longest latency intervals, often exceeding three decades. This finding is consistent with the concept that higher radiation doses induce more extensive and complex DNA damage, accelerating oncogenic transformation.

These results raise an important clinical question: should long-term survivors of cranial radiotherapy undergo routine neuroimaging surveillance decades after treatment? Given that latency periods cluster around 16–30 years depending on the radiation dose, our findings suggest that structured long-term MRI follow-up may warrant consideration in high-risk patients, particularly those exposed to high-dose cranial irradiation during childhood.

While universal lifelong screening may not be feasible or cost-effective, targeted surveillance strategies—such as initiating periodic MRI examinations 15–20 years after high-dose exposure and 25–30 years after lower-dose exposure—could facilitate earlier detection of RIMs, potentially allowing for less invasive treatment and improved outcomes. Prospective studies are needed to define optimal surveillance intervals and to balance early detection against resource utilization and patient burden.

### 4.5. Radiation Dose, WHO Grade, and Multiplicity

In addition to its effect on latency, radiation dose was strongly associated with both WHO grade and tumor multiplicity. High-dose irradiation was linked to a markedly higher proportion of high-grade meningiomas and a greater frequency of multifocal disease. These findings suggest a dose-dependent escalation not only in tumor initiation but also in biological aggressiveness and spatial dissemination.

The increased multiplicity observed after high-dose exposure may reflect widespread field cancerization within irradiated tissues, resulting in multiple independent tumor clones. Clinically, this has important implications for surgical planning and long-term management, as multifocal disease may limit the feasibility of complete resection and necessitate staged procedures or prolonged surveillance.

The studies included in this analysis span more than seven decades, during which radiotherapy techniques, treatment planning, fractionation strategies, and supportive care changed substantially. Earlier cohorts were often treated with less conformal techniques and broader radiation fields, whereas modern radiotherapy relies on more precise dose delivery and improved normal tissue sparing. These temporal differences may have influenced both the biological characteristics and the latency patterns of RIMs. However, detailed dosimetry and technical data were unavailable for most historical reports, precluding meaningful temporal subgroup analysis.

Modern radiotherapy techniques, including three-dimensional conformal radiotherapy, intensity-modulated radiotherapy, volumetric arc therapy, and proton therapy, have substantially improved target conformity and reduced unnecessary exposure of surrounding normal tissues. Whether these advances will reduce the long-term incidence of RIMs remains uncertain, particularly because many currently surviving patients were treated with historical techniques. Long-term follow-up of modern radiotherapy cohorts will be necessary to determine whether improved dose distribution translates into lower secondary tumor risk.

### 4.6. Clinical Implications

Taken together, our findings reinforce the concept that RIMs represent a biologically aggressive subset of meningiomas characterized by shorter latency after high-dose exposure, increased rates of high-grade histology, greater multiplicity, and higher recurrence risk. Awareness of these features is essential for clinicians involved in the long-term care of cancer survivors.

Early recognition of patients at risk, individualized follow-up strategies based on radiation dose and age at exposure, and careful surgical planning are critical to optimizing outcomes in this growing patient population.

### 4.7. Limitations

This study has several limitations. First, the available literature consisted predominantly of case reports, case series, and retrospective descriptive cohorts, resulting in heterogeneous and incomplete reporting and requiring variable-specific pooled analyses with different sample sizes. Second, because the analysis was based on published cases rather than population-based cohorts, recruitment bias and publication bias are likely, with unusual, aggressive, or recurrent tumors potentially being overrepresented. Third, survival bias must also be considered, as patients who lived long enough after irradiation to develop and be diagnosed with meningioma were inherently more likely to be captured in the literature. Fourth, detailed dosimetry, molecular, genetic and treatment-related data were unavailable for many historical cases, limiting more refined subgroup analyses. Finally, because the study design represented a pooled analysis of reported cases rather than a study-level comparative meta-analysis, conventional effect-size meta-analytic methods and formal heterogeneity measures were not applicable.

Nevertheless, the large number of included cases, the weighting of aggregated data by case counts, and consistent findings across multiple analyses strengthen the validity of our conclusions.

## 5. Conclusions

1. RIMs constitute a distinct subgroup of meningiomas with longer but dose-dependent latency, higher rates of high-grade histology, increased tumor multiplicity, and a substantial risk of recurrence compared with sporadic meningiomas.
2. The latency period of RIMs is significantly influenced by radiation dose and age at irradiation; higher doses and younger age at exposure are associated with earlier tumor development.
3. The histological aggressiveness of RIMs appears to be independent of the primary tumor type treated with radiotherapy, suggesting that ionizing radiation itself is the principal driver of malignant transformation.
4. Higher radiation doses used for treatment of the primary disease are associated with a higher WHO grade and increased tumor multiplicity of subsequent RIMs.
5. The observed latency patterns highlight the importance of long-term follow-up in patients previously exposed to cranial radiotherapy, particularly those irradiated during childhood.
6. These findings suggest that long-term neuroimaging surveillance may warrant consideration in survivors of cranial radiotherapy, with the timing of imaging potentially guided by radiation dose and age at exposure.

## Figures and Tables

**Table 1:** Clinical characteristics of patients with RIMs treated at our institution.

Case	Sex	Age at Irradiation	Age at RIM Diagnosis	Primary Disease	Latency Period, Years	Radiation Dose, Gy	Multiplicity	GTR	WHO Grade	Follow-Up, Months	Recurrence
**1**	M	2	22	NB	20	16	No	Yes	2	216	Yes
**2**	M	2.5	26	ALL	24	24	Yes	Yes	1	96	Yes
**3**	M	7	38	ALL	31	24	No	Yes	1	24	No
**4**	M	11	40	EPE	29	50	No	Yes	2	6	No

NB: neuroblastoma, ALL: acute lymphoblastic leukemia, EPE: ependymoma, and GTR: gross total resection.

**Table 2:** Data availability for variables included in the subgroup analyses.

Variable	Available Studies (*n*)	Available Cases (*n*)
**Latency period**	217	1303
**RIM WHO grade**	110	1019
**Radiation dose**	223	1478
**Primary tumor**	232	1746
**Recurrence**	102	440
**Multiplicity**	191	1047
**Age of irradiation**	203	980

**Table 3:** Classification of primary tumors treated with radiotherapy into predefined diagnostic categories used for subgroup analyses.

Tumor Category	Tumors List	Count, *n* (%)
**Skin disease** **of the head**	Tinea capitis, microsporosis, cutaneous angioma, skin hemangioma, basal cell carcinoma, unspecified scalp cancer, acne vulgaris, sebaceous naevus, unspecified scalp angioma, unspecified scalp lesion, breast cancer scalp metastasis, melanoma, unspecified primary skin carcinoma, abscess, and scalp infection	530 (48.05%)
**Acute leukemia**	ALL, AML, and other leukemia	378 (34.27%)
**Victim of a Nuclear Incident**	The explosion of the atomic bomb (Nagasaki) and exposure to Chernobyl irradiation	204 (18.50%)
**Other**	Otitis media, hypertrophic tonsils, schwannoma, Langerhans cell histiocytosis, CNS lymphoma, Graves ophthalmopathy, neurinoma, ganglioglioma, meningioma, AVM, unspecified CNS tumors, and an interventional embolization procedure for a brain aneurysm	180 (16.32%)
**Medulloblastoma**	Medulloblastoma	137 (12.42%)
**LGG**	Oligodendroglioma, astrocytoma, thalamic glioma, spongioblastoma, pontine glioma, pilocytic astrocytoma, optic nerve glioma, and unspecified LGG	98 (8.88%)
**Sellar/suprasellar tumor**	PitNET tumor, craniopharyngioma, and suprasellar germ cell tumor	62 (5.62%)
**Non-CNS lymphoma**	Hodgkin lymphoma, non-Hodgkin lymphoma, T-cell lymphoma, Burkitt lymphoma, and unspecified lymphoma	39 (3.54%)
**Ependymoma/subependymoma**	Ependymoma, subependymoma, and anaplastic ependymoma	33 (2.99%)
**Germ cell tumor**	Germ cell tumor and germinoma	19 (1.72%)
**Soft tissue tumor**	Ewing sarcoma, osteosarcoma, rhabdomyosarcoma, unspecified face tumor, carcinoma of the tongue, and unspecified soft tissue tumor	19 (1.72%)
**Head and Neck Tumor**	Parotid gland tumor, retroauricular tumor, thyroid tumor, nasal implant, facial angioma, oral squamous cell carcinoma, nasopharyngeal tumor, papilloma, and lineal tumor	11 (1.00%)
**HGG**	Anaplastic astrocytoma, glioblastoma, anaplastic oligoastrocytoma, anaplastic oligodendroglioma, and unspecified HGG	8 (0.73%)
**Other CNS embryonal tumor**	Olfactory neuroblastoma, atypical teratoid/rhabdoid tumor, ganglioneuroblastoma, and metastatic neuroblastoma	8 (0.73%)
**PNET**	PNET	8 (0.73%)
**Pineal tumor**	Pineal tumor	5 (0.45%)
**Ventricular tumor**	3rd ventricle tumor and unspecified ventricle tumor	4 (0.36%)
**Posterior fossa tumor**	Posterior fossa tumor other than medulloblastoma	3 (0.27%)

AML: acute myeloid leukemia, ALL: acute lymphoblastic leukemia, LGG: low-grade glioma, HGG: high-grade glioma, PNET: primitive neuroectodermal tumor, PitNET: pituitary neuroendocrine tumor, AVM: arteriovenous malformation, and CNS: central nervous system. For subgroup analyses, primary tumor categories with at least 30 reported cases were included to ensure sufficient statistical power and robustness of the results.

**Table 4:** Latency period, RIM WHO grade, and tumor multiplicity of RIMs according to the primary tumor category.

Primary Tumor Category		Latency Distribution		RIM WHO Grade			Multiplicity		
	*n*	Median Latency (IQR), Years	Range, Years	*n*	Low-Grade, *n* (%)	High-Grade, *n* (%)	*n*	Single, *n* (%)	Multiple, *n* (%)
**Medulloblastoma**	68	16 (11–23)	1.2–36	29	20 (69.0%)	9 (31.0%)	58	43 (74.1%)	15 (25.9%)
**LGG**	48	20 (12–26.3)	3–50	19	17 (89.5%)	2 (10.5%)	39	33 (84.6%)	6 (15.4%)
**Acute leukemia**	126	20.5 (16–26.7)	5–41	60	45 (75%)	15 (25%)	115	95 (82.6%)	20 (17.4%)
**Skin disease of the head**	393	36.3 (29.5–36.6)	2–63	100	88 (88%)	12 (12%)	380	325 (85.5%)	55 (14.5%)
**Sellar/suprasellar tumor**	57	20 (11–27)	1–36	24	16 (66.7%)	8 (33.3%)	47	40 (85.1%)	7 (14.9%)
**Ependymoma/** **subependymoma**	27	20 (12.2–26)	7–41	12	7 (58.3%)	5 (41.7%)	23	16 (69.6%)	7 (30.4%)
**Non-CNS lymphoma**	13	19 (11–23)	5–27.3	7	6 (85.7%)	1 (14.3%)	13	10 (76.9%)	3 (23.1%)
**Other**	15	15 (8.8–33)	2.5–59	8	6 (75.0%)	2 (25.0%)	13	9 (69.2%)	4 (30.8%)
**Victim of a Nuclear** **Incident**	68	9.4 (9.4–9.4)	9.4–9.4	88	84 (95%)	4 (5%)	-	-	-

Latency period is presented as the median with the interquartile range (IQR) and range due to a non-normal distribution. *n* reflects the number of cases with available latency data.

**Table 5:** WHO grade and tumor multiplicity according to the radiation dose category in RIMs.

WHO Grade	Radiation Dose Category			Total
	Low Dose	Intermediate Dose	High Dose	
**Low-grade, *n* (%)**	59 (10.9%)	233 (43.1%)	248 (45.9%)	540
**High-grade, *n* (%)**	19 (7.1%)	21 (7.9%)	226 (85.0%)	266
**Single, *n* (%)**	366 (86.3%)	117 (80.1%)	175 (73.5%)	658
**Multiple, *n* (%)**	58 (13.7%)	29 (19.9%)	63 (26.5%)	150

**Table 6:** Tumor recurrence according to WHO grade and the radiation dose category in RIMs.

	WHO Grade		Radiation Dose		
	Low-Grade	High-Grade	Low Dose	Intermediate Dose	High Dose
**No recurrence, *n* (%)**	143 (81.7%)	32 (48.5%)	131 (76.6%)	57 (72.2%)	134 (79.3%)
**Recurrence, *n* (%)**	32 (18.3%)	34 (51.5%)	40 (23.4%)	22 (27.8%)	35 (20.7%)

## Data Availability

The data supporting the findings of this study are available from the corresponding author upon reasonable request.

## Supplementary Materials

The following supporting information can be downloaded at: https://www.mdpi.com/article/10.3390/jcm15062356/s1, Table S1: JBI Checklist for case series. Table S2: List of all studies included in the pooled case analysis.

## Abbreviations

The following abbreviations are used in this manuscript:

RIM	Radiation-induced meningioma
SD	Standard deviation
IQR	Interquartile range
JBI	Joanna Briggs Institute
WHO	World Health Organization
